# Fifteen mRNA-lncRNA expression-based signature predicted the survival of late-staged head and neck squamous cell carcinoma

**DOI:** 10.1042/BSR20200442

**Published:** 2020-06-30

**Authors:** He Ren, Huaping Li, Ping Li, Yuhui Xu, Gang Liu, Liping Sun

**Affiliations:** 1 Faculty of Medical Instrumentation, Shanghai University of Medicine & Health Sciences, Shanghai 201318, China; 2 Department of Gynaecology and Obstetrics, Shanghai university of Medicine and Health Sciences Affiliated Zhoupu Hospital, Shanghai 201318, China; 3 Graduate School of Arts and Sciences, Columbia University, New York, NY 10027, U.S.A.

**Keywords:** head and neck squamous cell carcinoma, lncRNA, mRNA, signature, survival

## Abstract

**Background:** Gene expression is necessary for regulation in almost all biological processes, at the same time, it is related to the prognosis for head and neck squamous cell carcinoma (HNSCC). The prognosis of late-staged HNSCC is important because of its guiding significance on the therapy strategies.

**Methods:** In this work, we analyzed the relationship between gene expression and HNSCC in The Cancer Genome Atlas (TCGA) cohort, and optimized the panel with random forest survival analysis. Subsequently, a Cox multivariate regression-based model was developed to predict the clinical outcome of HNSCC. The performance of the model was assayed in the training cohort and validated in another three independent cohorts (GSE41614, E-TABM-302, E-MTAB-1328). The underlying pathways significantly associated with the model were identified. According to the results, patients of low-score group (median survival months: 27.4, 95% CI: 18.2–43) had a significant poor survival than those of high-score group (median survival months: 69.4, 95% CI: 58.7–72.1, *P*=2.7e-5), and the observation was repeatable in the other validation cohorts. Further analysis revealed that the model performed better than the other clinical indicators and is independent of these indicators.

**Results:** Comparison revealed that the model performed better than existing models for late HNSCC prognosis. Gene set enrichment analysis (GSEA) elucidated that the model was significantly associated with various cell processes and pathways.

## Background

Worldwide, head and neck squamous cell carcinoma (HNSCC) is one of the most lethal cancers. It was estimated that 108700 new cases and 56200 new deaths were reported in China, 2015 [1]. Although the clinical outcome of HNSCC, with survival rate less than 50% [2], is relatively poor compared with patients in early who are more curable, a large proportion of late-staged HNSCC patients survive over 2–5 years. Thus, it is necessary to distinguish these patients and adapt active or palliative therapy accordingly. Clinically, American Joint Committee on Cancer (AJCC) TNM staging system was widely used for prognosis for HNSCC [3], and differentiation grade is also an important pathological indicator. However, it is still not sufficient for clinical utilization.

In the past years, efforts have been devoted to unveil the underlying biological mechanisms that mRNAs influence the prognosis of HNSCC. For example, REG1 was shown to be significantly associated with survival in head and neck cancer [4]. The PD-L1 expression value was validated to have impact on many processes [5], and ERCC2 was reported to be associated with aggressive HNSCC [6]. Expression of C1GALT1 predicts the poor survival of head and neck cancer [7]. Recently, lncRNAs were gaining attention and microRNAs became hotspots as well. A large proportion of lncRNAs are expressed specifically in HNSCC, which show their potential to be prognostic biomarkers along with the other clinical indicators, and aberrant lncRNA expression has been found in many different kinds of cancers, including HNSCC [8–10]. However, due to the genetic and environmental heterogeneity of HNSCC, the prognostic value of single gene, including protein-coding and non-protein-coding genes, is not robust across cohorts. Therefore, multiple gene expression-based prognostic model has been emphasized upon during the past years. Mammaprint was developed using the expression of 70 genes [11], and has been reported to be an important guidance for breast cancer treatment.

In this work, we analyzed the gene expression microarray and next-generation based transcriptomic data of large HNSCC cohorts that were previously published in The Cancer Genome Atlas (TCGA), Gene Expression Omnibus (GEO) and Arrayexpress, screened the prognostic mRNAs and lncRNAs, optimized the panel and developed a prognostic model based on random forest survival method and Cox regression analysis. The model performance and robustness were evaluated, and underlying potential pathways associated with the model were identified.

## Materials and methods

### Sample and datasets involvement

In this work, HNSC gene expression datasets, derived from public and available GEO databases (http://www.ncbi.nih.gov/geo, accession number: GSE41613 [12]), TCGA (https://www.xena.ucsc.edu, accession: TCGA-HNSCC) and Arrayexpress (https://www.ebi.ac.uk/arrayexpress/, accession number: E-MTAB-1328 [13], E-TABM-302 [14]), were used. In each dataset, the samples diagnosed as late-staged HNSCC (TNM stage: IV) were selected, while samples with the other TNM stages or with incomplete clinical information were excluded.

### Data processing and model development

Raw data of TCGA were downloaded from UCSC Xena Browser (www.xena.ucsc.edu) and transformed to log2 (FPKM) values according to the provided method. Genes that did not detect reads in more than 70% samples were excluded in the following steps. The raw data of GSE41613 were downloaded from GEO (www.ncbi.nih.gov/geo) and normalized with R package ‘affy’ with rms method. The raw data of E-MTAB-1328 and E-TABM-302 were downloaded from Arrayexpress and processed with the same method as GSE41613. Univariate Cox regression analysis was used to evaluate the correlation of gene expression with patients’ overall survival information in TCGA dataset. Due to the large sample size compared with the other three, *P*-value <0.01 was considered to be statistically significant and used for further analysis. Random survival forests (RSFs) variable hunting algorithm was utilized to optimize the panel. The number of Monte Carlo iterations (nrep) was set as 100 in the RSF model, while the step size controlling values was set as 5 in the forward process (nstep). With a multivariable Cox regression model for the selected genes, the TCGA was used to estimate the risk score formula. The expression of these selected lncRNAs can be used to evolve formula by weighted by their estimated regression coefficients, as the following:

$$Score=\sum_{i=1}^{n}b_{i}x_{i}$$

Where, *b*_i_ indicates the coefficients and *x*_i_ represents the relative gene expression value.

### Gene set enrichment analysis

Gene set enrichment analysis (GSEA) [15] was conducted by GSEA software V2.2. As canonical representations of biological processes, ‘c2.cp.kegg.v6.2.symbols.gmt’ gene set was used for the enrichment analysis. Through Enrichment Map plug-in, we can observe the GSEA results in Cytoscape software V3.2.1. Gene sets were termed ‘enriched’ with which the false discovery rate (FDR) value was <0.05 after performing 1000 random sample permutations.

### Statistical analysis

By cutting off with the median score, patients were classified into a high- or low-risk group in each set on the basis of the risk score formula. To estimate the survival differences between the low- and high-risk groups, the Kaplan–Meier method with a log-rank test was chosen, and *P*<0.05 was considered as statistically significant in this step. Through univariate or multivariate Cox regression analysis, hazard ratios (HRs) and 95% CIs were evaluated. And the sensitivity and specificity of the prognosis of the score can be contrasted by receiver operating characteristic (ROC) curves using R package ‘pROC’. The correlation between scores and patients with different clinical features were measured through Student’s *t* test by categorizing the samples. The R V3.5.0 (www.rproject.org) program was employed to analyze amount data.

## Results

### Characteristics of datasets involved

In the present study, four independent sets of HNSCC subjects were involved. There were 296 HNSCC patients in the TCGA set, who had a mean follow-up time of 56 months. Data were as follows: 225 males (76.0%) and 71 females (24.0%); 145 (49.0%) patients were less than 60 years old and 151 (51.0%) patients over 60 years old; 92 (31.1%) patients were diagnosed as grade less than 4 and 204 (68.9%) were grade 4. All patients were diagnosed as TNM stage IV. In the E-TABM-302 set, the median follow-up period was 58 months. There were 58 male patients (93.2%), 40 (54.1%) less than 60 years old, and 51 patients diagnosed as grade 4. In the other two cohorts, E-MTAB-1328 and GSE41618, the median follow-up period were 57 and 26 months, respectively. The detailed information of samples involved is shown in Table 1.

**Table 1:** Clinical information of cohorts used in this study

Variables		TCGA	E-MTAB-302	E-MTAB-1328	GSE41618
Median follow-up (months)		56	58	57	26
Gender	Male	225	69	75	38
	Female	71	5	6	18
Age	60−	145	40	43	31
	60+	151	34	38	25
Grade	4−	92	23	24	NA
	4	204	51	57	NA

### Identification of prognostic lncRNAs and model development

The 296 HNSC patients in TCGA cohort were used for prognostic genes identification. The overall survival, tumor-free survival (TFS) and log2 transformed gene expression were correlated by using Cox univariate regression, and genes with *P*-value <0.01 in both TFS and OS analyses were retained. In this step, 78 genes significantly associated with overall survival and TFS were identified. However, the combination of these 78 genes were complex for clinical utilization, and thus it is necessary to optimize the panel where random forest variable hunting was implemented. In this step, 15 genes were selected, as shown in Table 2. The model was developed with Cox multivariate regression by correlating these 15 genes and overall survival information. The score is calculated as the following: score = (1.37289399*KLHDC7B) + (−0.327847579*EMC4) + (0.01524505*AHDC1) + (−0.646201302*PNPLA4) + (0.172110564*PEX11A) + (0.068037273*BRD4) + (0.731963077*NR1H2) + (−0.226687207*XCR1) + (−1.01799464*NFU1) + (−0.247036767*ORC6) + (0.468081738*AP000229.1) + (0.583104952*FAM53B) + (0.266319204*MIR4435-2HG) + (0.233982315*PLEKHG6) + (−0.333125317*ITFG1), where the gene symbols represent the expression values of corresponding genes. The positive coefficients suggest that these genes were oncogenes while the negative coefficients indicate that these genes were tumor suppressor genes.

**Table 2:** Genes in the risk score

Gene symbol	Ensembl IDs	Description	Coefficient
*KLHDC7B*	ENSG00000130487	Kelch domain containing 7B	1.37289399
*EMC4*	ENSG00000128463	ER membrane protein complex subunit 4	−0.327847579
*AHDC1*	ENSG00000126705	AT-hook DNA binding motif containing 1	0.01524505
*PNPLA4*	ENSG00000006757	Patatin-like phospholipase domain containing 4	−0.646201302
*PEX11A*	ENSG00000166821	Peroxisomal biogenesis factor 11 α	0.172110564
*BRD4*	ENSG00000141867	Bromodomain containing 4	0.068037273
*NR1H2*	ENSG00000131408	Nuclear receptor subfamily 1 group H member 2	0.731963077
*XCR1*	ENSG00000173578	X–C motif chemokine receptor 1	−0.226687207
*NFU1*	ENSG00000169599	NFU1 iron–sulfur cluster scaffold	−1.01799464
*ORC6*	ENSG00000091651	Origin recognition complex subunit 6	−0.247036767
*AP000229.1*	ENSG00000273492	Novel transcript	0.468081738
*FAM53B*	ENSG00000189319	Family with sequence similarity 53 member B	0.583104952
*MIR4435-2HG*	ENSG00000172965	MIR4435-2 host gene	0.266319204
*PLEKHG6*	ENSG00000008323	Pleckstrin homology and RhoGEF domain containing G6	0.233982315
*ITFG1*	ENSG00000129636	Integrin α FG-GAP repeat containing 1	−0.333125317

### Model performance evaluation

**Figure 1:**
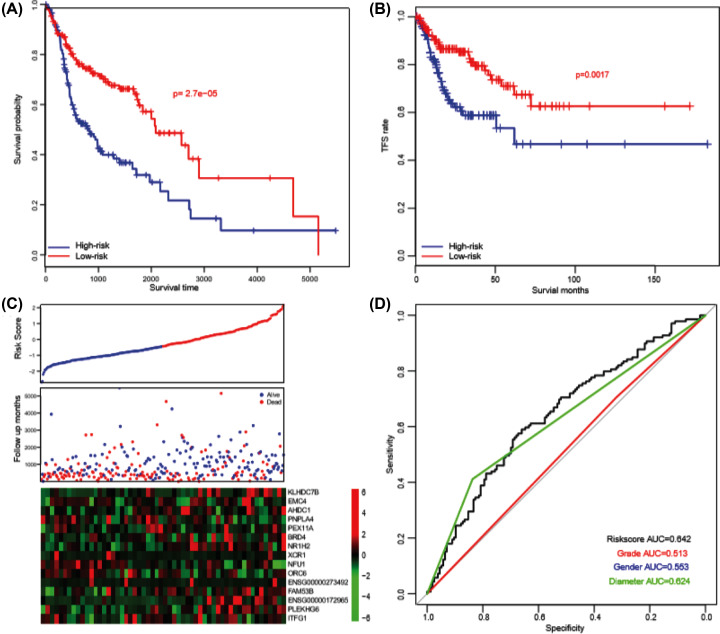
The model performance in training set The survival rate high-risk score is significantly lower in both overall survival (**A**) and TFS (**B**) pattern. High-risk group has a high death rate, low tumor suppressor gene and high expression of oncogenes (**C**). The survival ROC revealed that the risk score is a valuable marker of prognosis (**D**).

Patients were divided into a high-score group (score > −0.43, *n*=148) and a low-risk group (score ≤ 0.43, *n*=148) by taking the median risk score (−0.43) as the cut-off point. HNSCC patients with high-scores (median overall survival period: 27.4 months) had a significantly (*P*<0.001) shorter survival rate than these with lower scores (median overall survival: 69.4 months) (Figure 1A). The TFS pattern was also similar (*P*<0.01). As expected, the tumor suppressor genes were lowly expressed while the oncogenes were highly expressed in the high-score group, compared with the low-score group (Figure 1B). The prognostic value of the model was evaluated using the 2-year survival ROC curve (survival ROC), and the area under curve (AUC) was quantified and compared with clinical indicators (Figure 1C). The results reflected that the AUC of the score, grade, gender, primary tumor diameter was 0.642, 0.513, 0.553 and 0.624, respectively. Collectively, these results indicated that the model was a valuable prognostic biomarker.

### Robustness of the model

**Figure 2:**
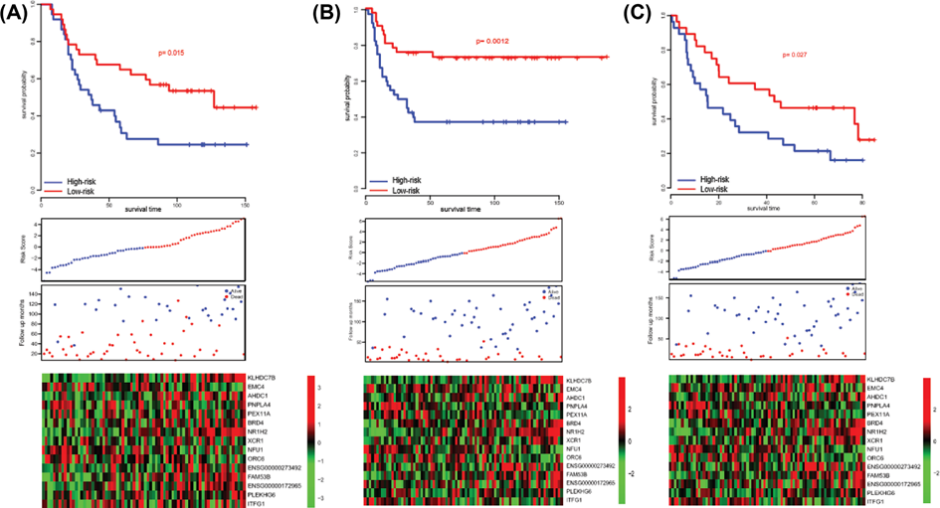
Performance validation of the model The high expression of oncogene, low expression of tumor suppressor gene and early incidence was reproducible in GSE41614 (**A**), E-TABM-302 (**B**), E-MTAB-1328 (**C**) datasets.

Since the model was developed on TCGA dataset, the high performance of the model may result from over fitness instead of its own characteristic. Thus, using exactly the same coefficients of the genes in the panel and the normalized genes’ expression values, the scores of samples in another three independent cohorts, E-TABM-302, E-MTAB-1328 and GSE41613, were calculated. The median score of each dataset was used to divide each cohort into high-score and low-score groups. As expected, the gene expression pattern of these 15 genes were similar to TCGA cohort and the high-score group had significantly worse survival than those in low-score group (*P*=0.015, 0.0012 and 0.027 for E-TABM-302, E-MTAB-1328 and GSE41613, separately, Figure 2). These results indicated that the model had great robustness for predicting the clinical outcome of late-staged HNSCC patients.

### Correlation between the model and clinicopathological features of HNSCC

**Figure 3:**
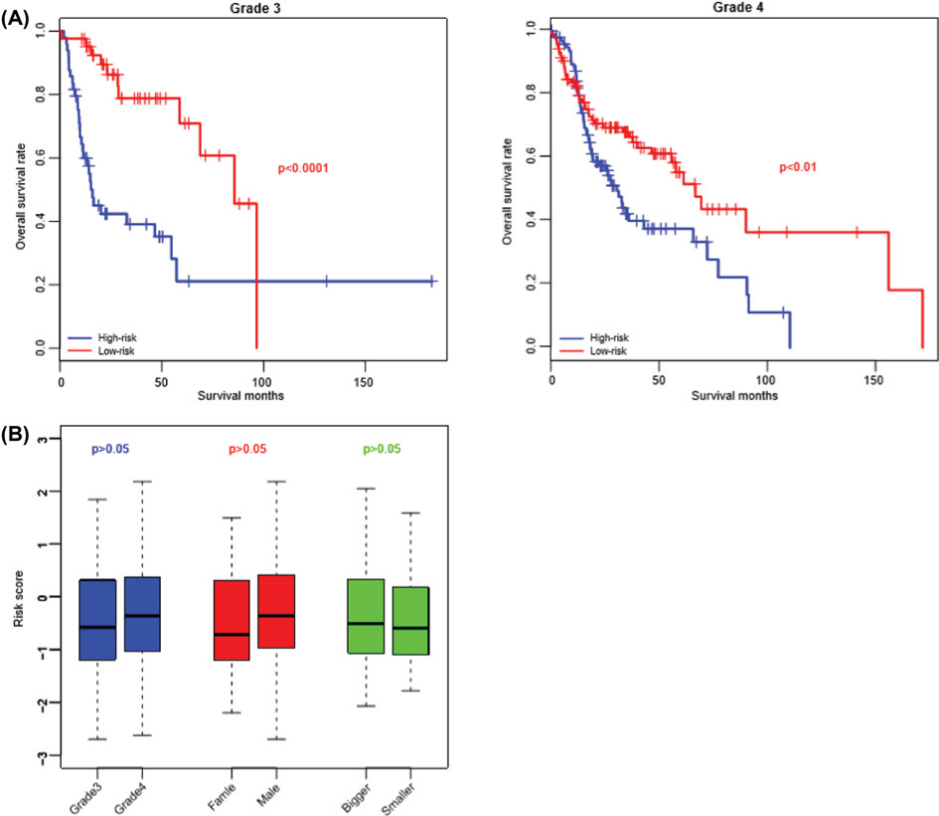
The prognostic value of risk score and clinical indicators The risk score has an unbiased prognostic value in Grade III and Grade (IV) patients (**A**), and independent from grade, gender and primary tumor size (**B**).

Patients can be equally grouped into two groups according to the clinical indicators (Grade 4 and 4−, female and male, primary tumor size >1 and <1 cm). And the differences of the scores were compared in the TCGA set. The results showed that the model was independent from these clinical indicators, as shown in Figure 3. Cox multivariate regression was implemented on TCGA cohort to evaluate the importance of the model, grade, gender and primary tumor size, and it was proved that primary tumor size and risk score were significantly associated with overall survival, while gender and grade were not (Table 3). Collectively, these results indicated that the model was independent from clinicopathological indicators and was important for late-staged HNSCC prognosis.

**Table 3:** Cox multivariate regression of risk score and other clinical information

	HR	95% CI	*P*
Grade	1.04	0.681–1.59	0.84508
Gender	0.93	0.60–1.45	0.7644
Tumor size	1.39	1.13–1.71	0.00137
Risk score	0.62	0.49–0.79	0.00017

### Comparison of the model and other models

**Figure 4:**
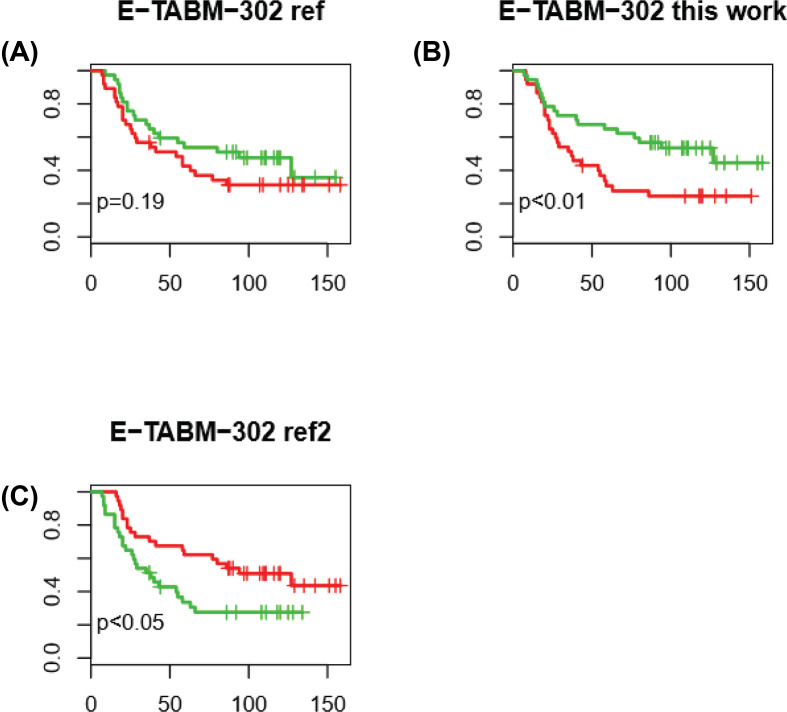
Comparison of the model with the other models The performance of the model was compared using E-TABM-302. The reference model is not statistically significant (**A**), our work (**B**), outperformed another model (**C**).

The performance of the model was compared with other models developed for HNSCC prognosis. Using the expression value of four genes, Rickman et al. developed a risk score model to predict the metastatic model [14]. Using seven lncRNAs and mRNAs, Zhang et al. developed a model for HNSCC prognosis [16]. Since both studies evaluated the performance with E-TABM-302 cohort, same as this work, the performance results were comparable. As expected, model in this work performed better than the other two models, the *P*-value is <0.01, <0.05 and >0.05, respectively (Figure 4A–C). The result indicated that our model was better for late-staged HNSCC prognosis.

## Discussion

Proteins were translated from mRNAs, and thus the abundance of mRNAs would have a huge impact on the protein functions, including carcinogenesis and cancer development, which had been validated by numerous reports. On the other hand, a lot of the genomic repertoire of non-protein-coding transcripts had been recognized as stochastic transcriptional product for a long time, including lncRNAs. And lncRNAs are remarkable for crucial function in cancer development and progression according to past reports. However, the prognostic value of lncRNAs for diseases has rarely been involved. The existing microarray gene expression data should be extracted from the GEO database. Additionally, we achieved lncRNA profiling to potential prognostic lncRNAs for HNSC. Then we analyzed the data of the lncRNA expression profiles. Recently, lncRNA combined mRNA model was emphasized for cancer biomarker development. However, such models’ applicability for late-staged HNSCC was still limited. The present study is the first to develop an lncRNA-mRNA model for late-staged HNSCC.

There were two lncRNAs in the model, ENSG00000273492 (also known as AP000229.1) and ENSG00000172965 (also known as MIR4435-2HG). Despite that there are currently no reports on the former lncRNA, prognostic value of MIR4435-2HG has been emphasized in other cancers, including hepatocellular carcinoma [17], colorectal cancer [18], lung cancer [19], gastric cancer [20] and glioblastoma [21]. The targets of MIR4435-2HG are known as miRNA-487a and β-catenin signaling, and thus this lncRNA can promote the proliferation of cells, while the exact mechanism is still unclear. KLHDC7B was shown to influence the breast cancer proliferation [22], BRD4 was reported to influence the immune cell infiltration in breast cancer [23] and promote the cMYC downstream genes’ transcription [24]. Similarly, the prognostic function of XCR1 was displayed in non-small cell lung cancer [25], hepatocellular carcinoma [26], and other cancers [27]. While reports regarding EMC4, AHDC1, PNPLA4 and PEX11A on the prognosis of HNSCC are limited, they suggested that the genes in the model were biologically functional.

## Abbreviations

AUC: area under curve
CI: confidence interval
GEO: Gene Expression Omnibus
GSEA: gene set enrichment analysis
HNSCC: head and neck squamous cell carcinoma
ROC: receiver operating characteristic
RSF: random survival forest
TCGA: The Cancer Genome Atlas
TFS: tumor-free survival
TNM: tumor node matastasis
